# Mobile Phone-Delivered Cognitive Behavioral Therapy for Insomnia: A Randomized Waitlist Controlled Trial

**DOI:** 10.2196/jmir.6524

**Published:** 2017-04-11

**Authors:** Corine HG Horsch, Jaap Lancee, Fiemke Griffioen-Both, Sandor Spruit, Siska Fitrianie, Mark A Neerincx, Robbert Jan Beun, Willem-Paul Brinkman

**Affiliations:** ^1^ Department of Intelligent Systems Delft University of Technology Delft Netherlands; ^2^ Department of Clinical Psychology University of Amsterdam Amsterdam Netherlands; ^3^ Department of Information and Computing Sciences Utrecht University Utrecht Netherlands

**Keywords:** insomnia, smartphone app, cognitive behavioral therapy, eHealth

## Abstract

**Background:**

This study is one of the first randomized controlled trials investigating cognitive behavioral therapy for insomnia (CBT-I) delivered by a fully automated mobile phone app. Such an app can potentially increase the accessibility of insomnia treatment for the 10% of people who have insomnia.

**Objective:**

The objective of our study was to investigate the efficacy of CBT-I delivered via the Sleepcare mobile phone app, compared with a waitlist control group, in a randomized controlled trial.

**Methods:**

We recruited participants in the Netherlands with relatively mild insomnia disorder. After answering an online pretest questionnaire, they were randomly assigned to the app (n=74) or the waitlist condition (n=77). The app packaged a sleep diary, a relaxation exercise, sleep restriction exercise, and sleep hygiene and education. The app was fully automated and adjusted itself to a participant’s progress. Program duration was 6 to 7 weeks, after which participants received posttest measurements and a 3-month follow-up. The participants in the waitlist condition received the app after they completed the posttest questionnaire. The measurements consisted of questionnaires and 7-day online diaries. The questionnaires measured insomnia severity, dysfunctional beliefs about sleep, and anxiety and depression symptoms. The diary measured sleep variables such as sleep efficiency. We performed multilevel analyses to study the interaction effects between time and condition.

**Results:**

The results showed significant interaction effects (*P*<.01) favoring the app condition on the primary outcome measures of insomnia severity (*d*=–0.66) and sleep efficiency (*d*=0.71). Overall, these improvements were also retained in a 3-month follow-up.

**Conclusions:**

This study demonstrated the efficacy of a fully automated mobile phone app in the treatment of relatively mild insomnia. The effects were in the range of what is found for Web-based treatment in general. This supports the applicability of such technical tools in the treatment of insomnia. Future work should examine the generalizability to a more diverse population. Furthermore, the separate components of such an app should be investigated. It remains to be seen how this app can best be integrated into the current health regimens.

**Trial Registration:**

Netherlands Trial Register: NTR5560; http://www.trialregister.nl/trialreg/admin/rctview.asp?TC=5560 (Archived by WebCite at http://www.webcitation.org/6noLaUdJ4)

## Introduction

Approximately 10% of the western adult population have chronic insomnia [1]. People with insomnia experience difficulties falling asleep, staying asleep, or both, and as a consequence they are sleep deprived during the day [2]. For example, insomnia is associated with low levels of concentration, greater fatigue, and impaired cognitive functioning [3-5]. Another consequence of insomnia is an increased risk of developing mental disorders such as depression and anxiety [6,7], or physical disorders such as diabetes and high blood pressure [8,9]. Insomnia also leads to societal costs such as reduced productivity, higher levels of sick leave, and more accidents [10].

One of the most common nonpharmacological treatments for insomnia is cognitive behavioral therapy (CBT-I). CBT-I is an effective treatment in either a face-to-face [11-13] or a self-help format [14,15]. Recently it has become more common to offer these self-help formats via the Internet. A recent meta-analysis [16] demonstrated that Internet-delivered CBT-I showed large treatment effects (Cohen *d*=1.0) on the Insomnia Severity Index (ISI). In addition to the efficacy of computerized CBT-I (CCBT-I), this format has multiple other advantages over face-to-face treatments. Potentially it can save costs, because less therapist time is needed, and the treatment can be offered to a larger number of people who can go through the treatment in their own time.

Until now, studies on computerized treatments have been mostly limited to Web-based treatments. A possible next step is delivering CCBT-I via a mobile phone app. CCBT-I delivered via a mobile phone has similar advantages to existing CCBT-I, such as wide and easy accessibility, reduced stigma, and greater cost-efficiency [17], but it could potentially exceed those advantages because mobile phones are portable. People carry their phones with them all the time and they are ubiquitous, unobtrusive, and intimate. Therefore, an effective app-based treatment for insomnia would increase the possible coverage for CBT-I. Furthermore, mobile phones are rich in sensors, computationally powerful, and remotely accessible, which provides opportunities for personalization, ecological momentary access, and real-time tracking [18,19].

In the domain of sleep, several kinds of sleep apps have been studied. For example, there is an app that unobtrusively increases awareness of sleep hygiene recommendations [20], an app that applies active sleep sampling for measuring sleep [21], a social app that shares time in bed based on alarm usage [22], and an app that supports and was used alongside of face-to-face CBT-I. To the best of our knowledge, however, no studies have evaluated the efficacy of stand-alone CCBT-I apps. To bridge this gap, we conducted a randomized controlled trial to compare a group using a CCBT-I-based app with a waitlist control group. We expected that this app would have an ameliorating effect on insomnia severity and sleep impairment compared with the waitlist control group, assessed by a sleep diary.

The app offered a sleep diary, a relaxation exercise, sleep restriction exercise, and sleep hygiene and education. Since sleep restriction is seen as the most effective exercise [23,24], it was the main focus of the app. The goal was to demonstrate the app’s efficacy in a sample of patients with relatively mild insomnia in order to test the proof of principle before investigating it in a more severely affected population.

## Methods

This study had a between-participants design with 2 arms: a waitlist condition and an intervention condition, with preintervention, postintervention, and 3-month follow-up measures.

### Participants

**Figure 1:**
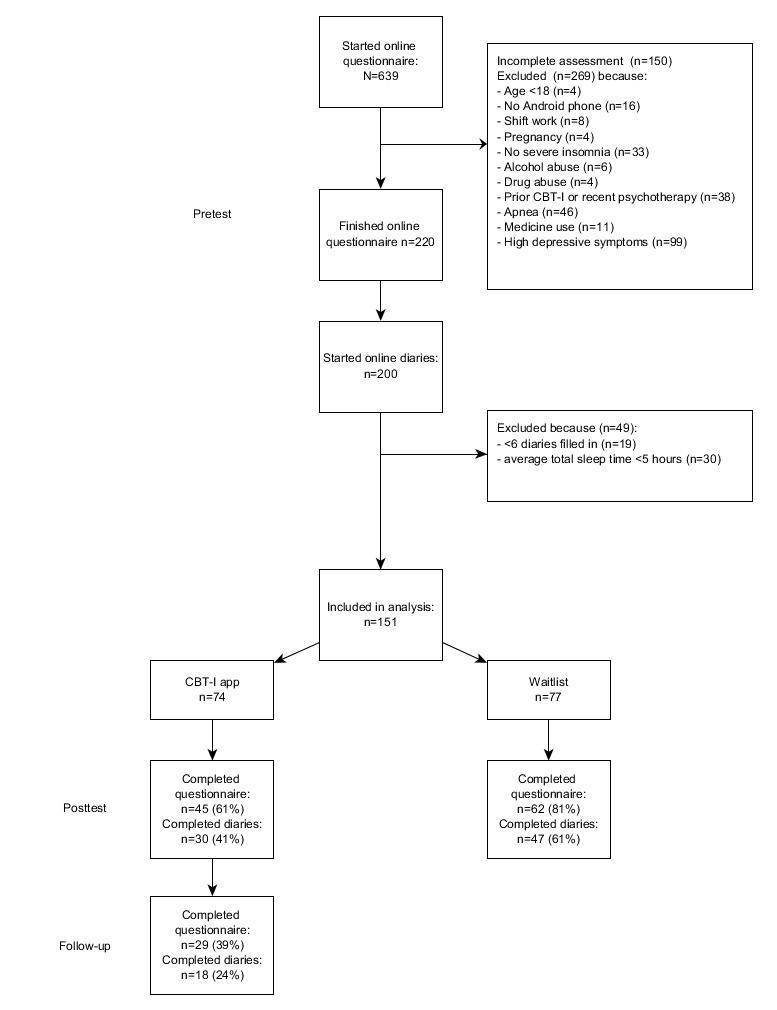
Consolidated Standards of Reporting Trial (CONSORT) flow diagram of recruitment, reasons for exclusion, and experimental compliance. CBT-I: cognitive behavioral therapy for insomnia.

We recruited participants from August 15 to October 21, 2015, via websites, social media, online advertisements, flyers, and a press release in the Netherlands. An initial group of 639 interested individuals completed an informed consent form and started the online questionnaire. Of this group, we excluded 269 people based on the inclusion and exclusion criteria (see Figure 1; Multimedia Appendix 1 [25]). Inclusion criteria were (1) difficulty with initiating or maintaining sleep for at least 30 minutes a night, for at least 3 nights a week, for at least 3 months, causing clinically significant distress or impairment in daily functioning, in accordance with the criteria for a *Diagnostic and Statistical Manual of Mental Disorders* (DSM-5) diagnosis of insomnia [2], (2) stable medication use, (3) aged ≥18 years, and (4) a valid email address, connected to the Internet, and in possession of an Android mobile phone (operating system version 4.1 or higher). Exclusion criteria were (1) ISI score [26] <7, (2) previous treatment with CBT-I (3) having started other psychotherapy in the last 6 months, (4) self-reported diagnosis of schizophrenia or psychosis, (5) alcohol or marijuana abuse (>3 glasses of alcohol a day for at least 21 days a month, or use of marijuana more than once a week), (6) possible sleep apnea (determined with a subscale of the SLEEP-50 questionnaire; cut off ≥15 [27]), (7) shift work, (8) pregnant or breast-feeding, (9) symptoms of depression (determined with a subscale of the Centre for Epidemiological Studies Depression [CES-D] scale [28,29]; cutoff ≥27), or (10) total sleep time ≤5 hours on average as reported in a consecutive 7-day sleep diary prior to the experiment. We measured all inclusion and exclusion criteria, except the last one, using an online questionnaire asking the participants directly about the criteria (see Multimedia Appendix 2).

### Intervention

**Figure 2:**
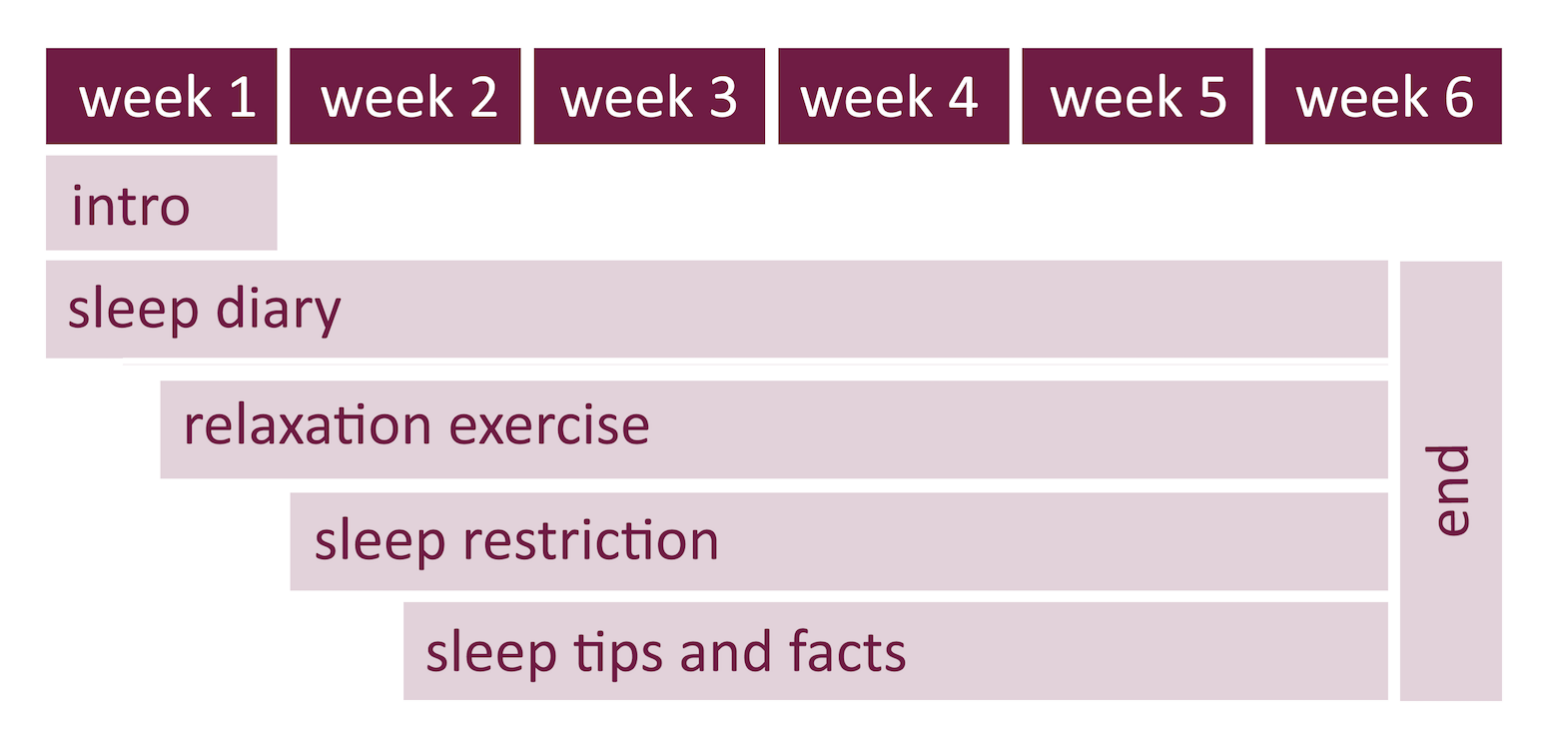
Sleepcare app treatment protocol.

The Sleepcare app [30,31] was based on previously published protocols (eg, [32,33]) and followed a talk-and-tool design principle, which is based on the idea that people interact symbolically and physically with their environment (see [34] for more details on the rationale and design decisions). The app packaged a sleep diary, a relaxation exercise, sleep restriction exercise, and sleep hygiene and education (Figure 2). The app offered these exercises in Dutch, adjusted them to the participant, and reminded participants to perform the exercises. The basic program duration was 6 to 7 weeks, depending on a participant’s adherence. For example, if a participant had filled out <6 sleep diaries since starting the app, the app explained to the participant that the sleep restriction exercise could start only after they had completed 6 diaries. If they had completed <6 diaries, the introduction of the sleep restriction exercise was postponed until participants met this prerequisite. The app was fully automated and did not require any input from therapists or a human administrator. Automatic warnings were built in when participants slept for <5 hours on average. The first warning appeared after 5 days and warned against activities such as driving a car while feeling sleepy. Follow-up warnings also included a referral to the general practitioner, and the app automatically stopped the sleep restriction exercise.

**Figure 3:**
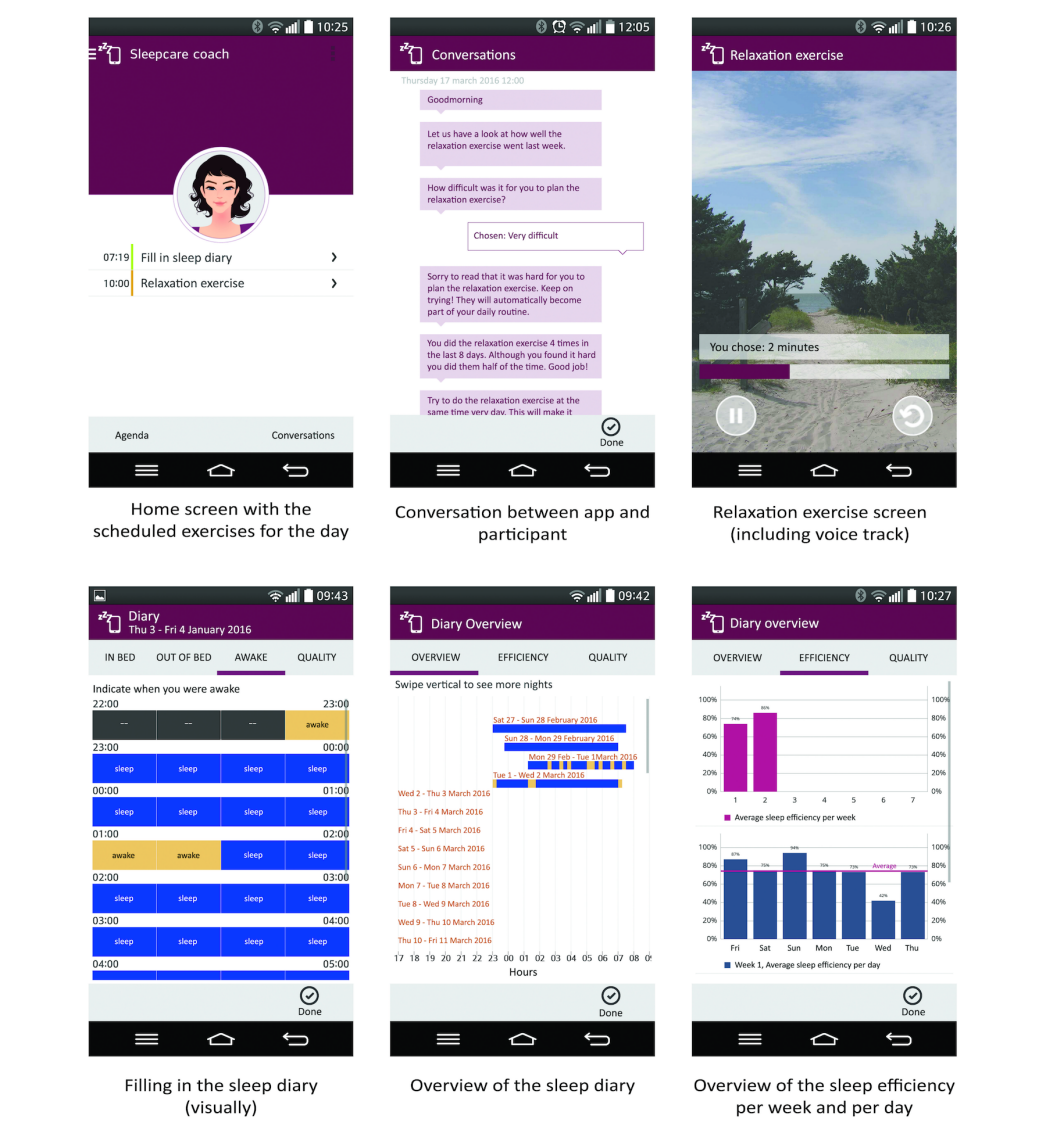
Mock-up screenshots of the Sleepcare app translated from Dutch.

The app consisted of a home screen that displayed the scheduled exercises for that day. Furthermore, there was a menu, a calendar, and a conversation screen (Figure 3). The menu provided access to all components of the app and the CBT-I exercises. The calendar displayed all the scheduled activities for the whole 7 weeks, which the participants could browse through at any time. The app interacted with the participants via dialogues on the conversation screen.

#### Conversations

The conversation screen (Figure 3) displayed the dialogues between the app and the participants, which were inspired by face-to-face consultations. New exercises were introduced, and the progress of the participants was evaluated. Typically, the app gave information and asked multiple-choice questions. Participants could open new conversations only after the previous conversation was finished. Conversations were initiated by the app based on a participant’s adherence and progress. For example, if a relaxation exercise was done <3 times within 4 days of its introduction, a conversation would start to address the participant’s adherence. Additionally, the app started conversations based on a participant’s progress; for example, after the participant had filled out the sleep diary for a week, the app calculated and showed weekly sleep efficiency averages in an evaluation conversation. A detailed description of the underlying design principles can be found in Beun et al [34].

#### CBT-I Exercises

##### Sleep Diary

The sleep diary was a visual translation of the core Consensus Sleep Diary [35] consisting of 4 screens asking participants to fill in their bedtimes and wake times, and their subjective sleep quality. The sleep diary could only be filled out for the previous night. Connected to the sleep diary was the sleep overview, which gave a visual summary of the participant’s sleep.

##### Relaxation Exercise

Relaxation exercises have a long history for treating insomnia. Based on the idea that hyperarousal is a main determinant of insomnia, relaxation exercises are the designated treatment [36]. The relaxation exercise was a progressive muscle relaxation exercise of varying durations, ranging from 1 to 16 minutes. The participants could choose the length of the exercise themselves before starting the exercise. By being offered short exercises, participants were able to gradually develop the habit of relaxing. The participants were guided by a voice track, which told them which muscles to contract and when to relax. The app advised participants to do the relaxation exercise once a day, although participants could do the exercises as often as they wanted [37].

##### Modified Sleep Restriction

Sleep restriction is seen as the most effective exercise [23,24], and therefore was the main focus of the app. After a week, the app introduced the modified sleep restriction exercise, on condition that participants had filled ≥6 sleep diaries and had an average sleep efficiency of <85%. We developed an algorithm to calculate the ideal and maximum time in bed for that specific participant based on the average sleep times of the previous week (FGB, unpublished data, 2016). The algorithm used rules such as (1) the ideal time in bed is equal to the average time in bed, (2) the maximum time in bed is always at least 1 hour less than the average time in bed, (3) the advised time in bed is never <5 hours [38], and (4) the advised time in bed lies between average total sleep time and average time in bed. Participants had the opportunity to negotiate their sleep time. The app first suggested that the participant abide by the ideal time in bed. The participant was then given the option to accept that time in bed, to negotiate longer time in bed up to the calculated maximum time in bed, or to refrain from sleep restriction completely. Participants were allowed to negotiate about and refrain from the sleep restriction exercise in order to enhance self-empowerment, set realistic goals, and thereby increase adherence. Every week the app evaluated the adherence and effect of the sleep restriction exercise. When participants’ sleep efficiency was >85%, they were allowed 15 minutes extra in bed. When a participant’s average sleep efficiency was <85%, the app suggested the same or a further restriction of 15 minutes, depending on a participant’s adherence. We used a modified sleep restriction protocol because we assumed this would increase the possibility of completing the restriction exercise, on the basis that more lenient sleep restriction is better than no sleep restriction at all.

##### Sleep Hygiene and Education

Sleep hygiene and education consist of lifestyle recommendations and knowledge about sleep. As a single-component intervention, it lacks efficacy [13]. However, it lays the foundation for CBT-I, since it increases participants’ knowledge about sleep and the factors influencing sleep. By increasing understanding, sleep hygiene and education increase the efficacy of the other CBT-I exercises, and are therefore included in many multicomponent interventions [36]. Sleep hygiene and education information was presented on different screens as tips and facts in text format. The tips were divided into 3 categories: (1) food and drink, (2) bedroom, and (3) behavior. “Use your bedroom only for sleeping, not for working” was an example of a bedroom tip. The sleep facts were categorized into 8 groups: (1) sleep cycles, (2) amount of sleep, (3) age, (4) animals, (5) disorders, (6) causes, (7) sleep medicine, and (8) fun facts. An example of a fact about age is “The amount of sleep a person needs is age dependent.”

#### Persuasive Strategies

We implemented various kinds of persuasive strategies to support the participants’ adherence. First, the app sent notifications for both the exercises and the conversations. So, for a scheduled exercise such as filling in the sleep diary, the app sent the participants a notification. For unfinished conversations, additional reminders were sent every day at noon. Second, the app provided room for negotiation about the sleep restriction assignment as described earlier. Furthermore, the app was designed to be easy to use and attractive, to improve adherence [31,39].

### Measurements

#### Questionnaire Measures

##### Primary Measure

We measured the severity of insomnia with a Dutch translation of the widely used ISI. This is a 7-item questionnaire with scores ranging from 0 (no insomnia) to 28 (severe insomnia). A cutoff score of 7 determined relatively mild insomnia [26]. We set the cutoff score at this level instead of the clinically more relevant cutoff score of 10 [40] because the goal of this study was to demonstrate the feasibility of the app in a sample of patients with at least subclinical levels of insomnia.

##### Secondary Measures

The Pittsburgh Sleep Quality Index (PSQI) is a self-rating scale that measures sleep disturbances over a 1-month period [41]. The PSQI consists of 19 items with scores from 0 to 3 evaluating 7 subdomains. The scores of these subdomains are summed to calculate a global score ranging from 0 to 21. A global score >5 indicates severe impairment in at least two domains, or moderate impairment in at least three domains.

We measured dysfunctional beliefs with the Dutch translation of the brief Dysfunctional Beliefs and Attitudes about Sleep (DBAS-16) scale [42]. The DBAS-16 consists of 16 statements with scores from 0 to 10 to indicate how much people agree with the statement. The average is calculated so that the total score ranges from 0 (no dysfunctional beliefs) to 10 (severe dysfunctional beliefs).

Anxiety symptoms were assessed with the 7 anxiety items of the Dutch version of the Hospital Anxiety and Depression Scale (HADS) [43,44]. The summed score ranges from 0 (no symptoms of anxiety) to 21 (severe symptoms of anxiety).

Depressive symptoms were measured using a Dutch translation of the CES-D scale. The CES-D consists of 20 items with scores ranging from 0 to 3, which are summed, with higher scores indicating more depressive symptoms [28,29].

#### Diary Measures

We used an online Dutch translated version of the consensus sleep diary [35]. Participants filled out the sleep diary for 7 days. In the diary they recorded the time they went to bed, the time they tried to go to sleep, their time of final awakening, their time out of bed, sleep onset latency, wake after sleep onset, terminal wakefulness, number of awakenings, sleep quality (1 = “very bad” to 10 = “very good”), and use of sleep medication. From these variables, we calculated the time in bed (time in bed=final arising time–time of going to bed), sleep time (total sleep time=time in bed–sleep onset latency–wake after sleep onset–terminal wakefulness), and sleep efficiency (sleep efficiency=[total sleep time/time in bed]×100). Sleep efficiency was the second primary measure in this trial.

#### Process Measures

We measured motivation to use the app with the Situational Motivation Scale [45] and acceptance of the app with the unified theory of acceptance and use of technology [46]. The focus of this paper is on the outcome measures, so we do not include the results of the process measures.

### Procedure

Participants gave online informed consent and filled out the questionnaire addressing the inclusion and exclusion criteria, demographic information, and the outcome measures. The participants who met the study criteria received a link to an online sleep diary by email for 7 successive days. Emails for the diary were sent at 6.00 AM, and a reminder email was sent at 10.00 AM. We excluded participants who reported an average total sleep time of <5 hours and then randomly assigned the others to either the app or the waitlist condition. Randomization was carried out by a third party who was not part of this study. They used an online tool [47] to generate blocks of 20 participants. The list of the randomization sequence was kept in a locked office cupboard by the third party. After participants were assigned to a condition, participants and the principal investigator (CH) were no longer blinded to the condition allocation.

Then, 3 weeks after starting with the app or the waitlist condition, all participants received an interim measurement consisting of the ISI and DBAS-16, supplemented with questions regarding motivation (Situational Motivation Scale) and app acceptance for the app group. These interim measures are not reported in this paper. Both groups received a postintervention questionnaire, 7 weeks after random assignment, consisting of all the outcome measures (ISI, PSQI, DBAS-16, CES-D, and HADS) and a 7-day diary. In addition, participants in the app group received questions regarding the effect and utility of the app, which are not reported in this paper. After completing the diary, participants in the waitlist condition received the app. Participants in the app condition additionally received a 3-month follow-up questionnaire and diary.

The study was approved by the internal Ethical Review Board of the University of Amsterdam, and was registered with the Netherlands Trial Register (NTR5560).

### Statistical Analysis

#### Required Statistical Power

To our knowledge, this study is the first large-scale randomized controlled trial to study an app to treat chronic insomnia, so expected effects were unknown. Earlier research about Web-based CBT-I found a Cohen *d*>1.0 [48,49]. We were uncertain whether these large effects could also be obtained by an app, so we anticipated an average effect. A power calculation for a mixed analysis of variance design (effect: *f*^2^=0.15, power 80%, alpha=.05) indicated that a total of 90 participants were needed to detect a potential difference between the 2 conditions. As a meta-analysis showed that on average 50% of people adhere to technology-mediated insomnia treatment [50], the goal was set to include 180 participants.

#### Analyses

We tested the effects of the intervention using multilevel analyses, which allows for the inclusion of participants with 1 measurement and therefore is appropriate for intention-to-treat analyses [51]. Models were built in R version 3.1.3 (The R Foundation) to explore within-group (time), between-group (condition: app vs waitlist), and interaction (time × condition) effects. Model 0 is the basic model and includes only the participants as a random intercept. Model 1 adds the fixed factor time to model 0. Model 2 was built on model 1 and adds the condition as a fixed effect. Finally, model 3 adds the interaction effect between time and condition. Models 4 and 5 concern the premeasurement and follow-up data. Model 4 is the null model that includes only the participants as a random intercept. In model 5 time is added. Since there were no follow-up measurements for the waitlist, condition is not included. Dropout analyses for the postmeasurements showed that age, sleep quality, and terminal wakefulness were associated with nonresponse in the app condition. In the waitlist condition, number of awakenings was related to nonresponse. Dropout analyses for the follow-up measurements showed that terminal wakefulness was associated with nonresponse. Therefore, they were added as covariates in all models in the multilevel regression analyses [51]. We calculated chi-squares for the various models to compare the ability of the models to fit the data. Furthermore, we calculated *R^2^* values to indicate the level of variance explained by the level-1 variables [52]. *R^2^* values of .10 indicate a small effect, *R^2^*=.30 indicates a medium effect, and *R^2^*=.50 indicates a large effect [53].

To enhance comparability with other studies, we calculated between-group Cohen *d* values. Table 1 shows the means and effect sizes based on an imputed dataset. First, we used multiple imputation in IBM SPSS version 22 (IBM Corporation) to insert missing cases [54]. Data from 41-44 participants (27.2%-29.1%) were missing for the outcome questionnaires. Diaries were missing from 76 of the 151 participants (50.3%). The follow-up measurements were not imputed due to a large amount of missing data. For imputation, we used the pre- and post measures of the ISI, PSQI, DBAS-16, CES-D, HADS, sleep quality, sleep onset latency, wake after sleep onset, number of awakenings, time in bed, terminal wakefulness, total sleep time, and sleep efficiency, next to sex and age. With a predictive mean matching procedure, 10 separate datasets were generated. The values in Table 1 are based on these imputed datasets. Second, we calculated Cohen *d* values by dividing the difference in change scores by the pooled standard deviation of that change score (*d*=[mean_change score waitlist_–mean_change score app_]/SD_pooled_), whereby mean_change score_=mean_pre_–mean_post_. We calculated within-group Cohen *d* values using the pre- and post scores per condition and the pooled standard deviation (*d*=[mean_pre_–mean_post_]/SD_pooled_). Additionally, we calculated within-group Cohen *d* values with the pre- and follow-up scores per condition and the pooled standard deviation (*d*=[mean_pre_–mean_follow-up_]/SD_pooled_) (Table 2). A Cohen *d* of 0.20 indicates a small effect, 0.50 a moderate effect, and 0.80 a large effect [53].

**Table 1:** Observed baseline and imputed posttest means and effect sizes.

Measure		Group	Score, mean (SD)		Cohen *d*		95% CI (change scores)
			Baseline	Posttest	Within group	Between groups	
**Questionnaire**							
	ISI^a^	WL^b^	16.4 (3.3)	13.2 (4.5)	0.80	–0.66	–0.99 to –0.33^c^
		App	16.4 (3.1)	9.9 (4.9)	1.33		
	DBAS-16^d^	WL	5.2 (1.3)	4.8 (1.6)	0.24	–0.15	–0.47 to 0.17
		App	5.3 (1.3)	4.7 (1.4)	0.41		
	CES-D^e^	WL	15.0 (5.8)	15.5 (9.5)	–0.06	–0.94	–1.28 to −0.61^c^
		App	16.5 (6.0)	11.0 (5.6)	0.98		
	HADS^f^	WL	5.6 (3.1)	6.2 (3.8)	–0.15	–0.75	–1.08 to −0.42^c^
		App	6.1 (3.0)	4.1 (2.5)	0.81		
	PSQI^g^	WL	10.6 (2.8)	9.7 (2.9)	0.32	–0.77	–1.10 to −0.44^c^
		App	11.0 (2.8)	7.4 (3.3)	1.09		
**Diary**							
	Sleep efficiency	WL	77.0 (8.2)	78.3 (7.6)	–0.16	0.71	0.37 to 1.04^c^
		App	77.6 (7.3)	84.8 (5.3)	–1.37		
	Time in bed	WL	500 (46)	513 (34)	–0.32	–0.55	–.88 to −0.22^c^
		App	506 (44)	495 (31)	0.36		
	Total sleep time	WL	386 (49)	401 (47)	–0.32	0.24	–0.08 to 0.56
		App	393 (52)	421 (37)	–0.74		
	Sleep onset	WL	31 (21)	30 (19)	0.06	–0.45	–0.77 to −0.12^c^
		App	33 (20)	20 (12)	1.01		
	Wake after sleep onset	WL	44 (30)	44 (25)	–0.02	–0.70	–1.03 to −0.36^c^
		App	45 (32)	24 (11)	1.79		
	Terminal wakefulness	WL	37 (20)	37 (15)	0.02	–0.26	–0.58 to 0.07
		App	35 (22)	29 (13)	0.52		
	Number of awakenings	WL	2.22 (1.14)	2.14 (1.08)	0.07	–0.38	–0.70 to −0.05^c^
		App	1.94 (0.99)	1.46 (1.14)	0.42		
	Sleep quality	WL	2.93 (0.52)	3.10 (0.55)	–0.33	0.29	–0.04 to 0.61
		App	2.97 (0.41)	3.33 (0.54)	–0.67		

^a^ISI: Insomnia Severity Index.

^b^WL: waitlist condition.

^c^The confidence interval does not contain zero, meaning the effect apparently exists.

^d^DBAS-16: brief Dysfunctional Beliefs and Attitudes about Sleep.

^e^CES-D: Centre for Epidemiological Studies Depression.

^f^HADS: Hospital Anxiety and Depression Scale.

^g^PSQI: Pittsburgh Sleep Quality Index.

**Table 2:** Completers sample: baseline, posttest, and follow-up mean scores for the app condition and within-group effect sizes for the baseline (pre) to follow-up measurements.

Measure		Baseline score mean (SD)	Posttest score mean (SD)	Follow-up score mean (SD)	Cohen *d*
**Questionnaire**					
	ISI^a^	16.4 (3.1)	9.8 (4.8)	10.0 (5.3)	1.20
	DBAS-16^b^	5.3 (1.3)	4.7 (1.4)	4.3 (1.8)	0.58
	CES-D^c^	16.5 (6.0)	10.3 (5.3)	11.0 (7.2)	0.75
	HADS^d^	6.1 (3.0)	4.0 (2.4)	4.3 (2.8)	0.67
	PSQI^e^	11.0 (2.8)	7.6 (3.1)	9.1 (3.6)	0.53
**Diary**					
	Sleep efficiency	77.6 (7.3)	83.8 (8.3)	83.8 (10.9)	–0.57
	Time in bed	506 (44)	496 (50)	483 (39)	0.57
	Total sleep time	393 (52)	417 (62)	403 (57)	–0.17
	Sleep onset	33 (20)	22 (14)	21 (15)	0.80
	Wake after sleep onset	45 (32)	27 (21)	25 (24)	0.84
	Terminal wakefulness	35 (22)	31 (22)	35 (36)	0.13
	Number of awakenings	1.94 (0.99)	1.58 (1.10)	1.75 (1.44)	0.01
	Sleep quality	2.97 (0.41)	3.38 (0.51)	3.41 (0.60)	–0.74

^a^ISI: Insomnia Severity Index.

^b^DBAS-16: brief Dysfunctional Beliefs and Attitudes about Sleep.

^c^CES-D: Centre for Epidemiological Studies Depression.

^d^HADS: Hospital Anxiety and Depression Scale.

^e^PSQI: Pittsburgh Sleep Quality Index.

## Results

### Baseline Characteristics of the Sample

We randomly assigned 151 participants to the app (n=74) or a waitlist condition (n=79). Participants had a mean age of 39.66 years (SD 13.44; range 18-80). Of the total 151 participants, 94 were female (62.3%). Table 3 shows demographic information about the participants, as well as the randomization check. At baseline, the groups did not differ significantly on any demographic characteristics (all *P* values >.05).

### Efficacy: Intention-to-Treat Analyses

**Figure 4:**
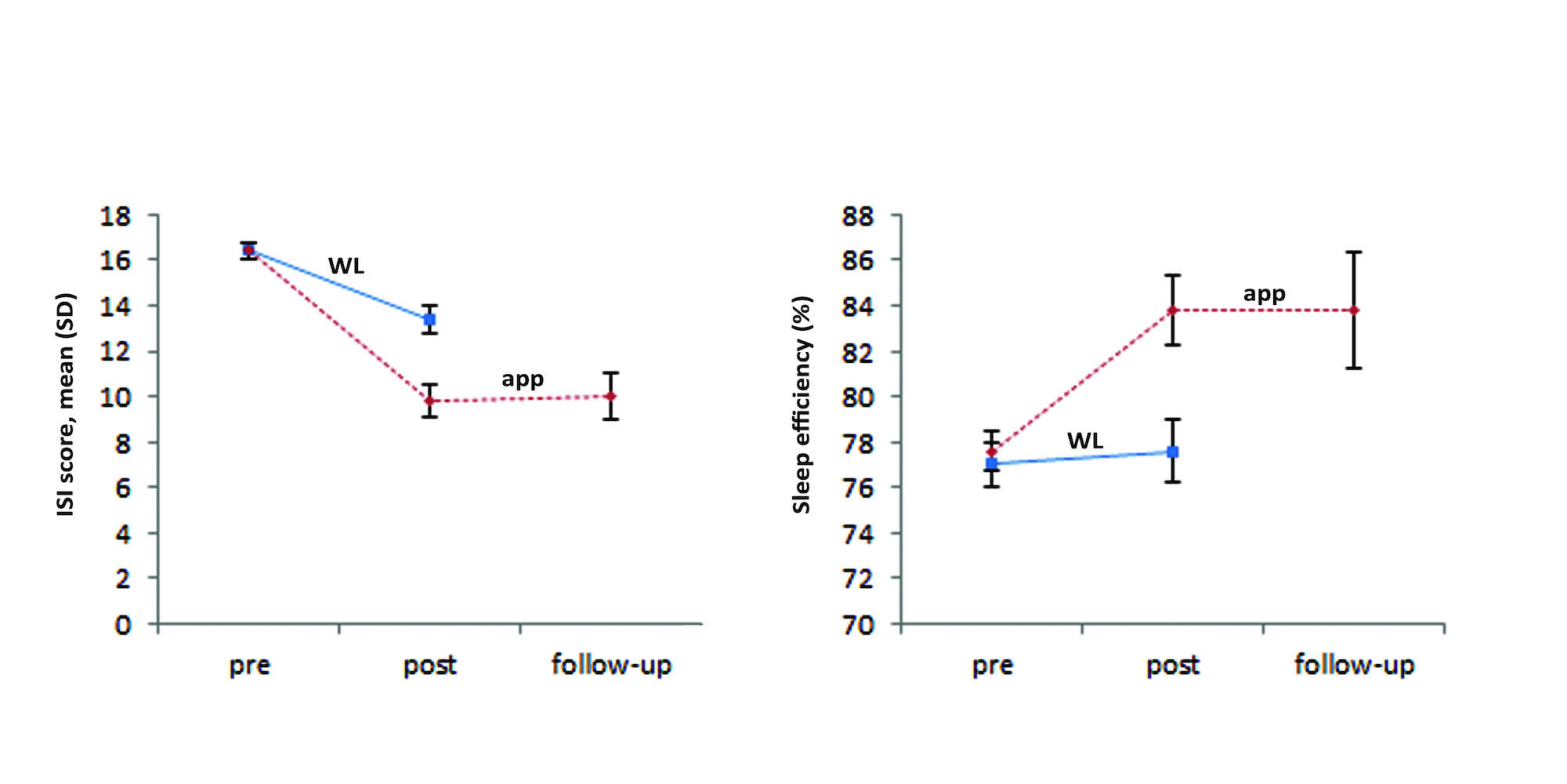
Completers sample: Insomnia Severity Index (ISI) scores and sleep efficiency of the Sleepcare mobile phone app group compared with the waitlist (WL) control group at baseline, posttest, and 3-month follow-up. Error bars represent standard error.

Table 1 displays the mean scores for all the outcome measures and corresponding Cohen *d* values for the baseline and postmeasurements. Table 2 displays the mean scores for the follow-up measures. Figure 4 depicts the scores for the main outcome measures ISI and sleep efficiency. Table 4 and Table 5 present the results of the multilevel analyses. Table 4 shows the coefficients for model 3 and their standard errors, and whether the coefficients were significant. Table 5 shows whether there was a significant difference between the models (χ^2^) and the level of variance explained (*R^2^*). The multilevel analyses showed significant interaction effects between time and condition on the primary outcome measures ISI (*d*=–0.66) and sleep efficiency (*d*=0.71) at posttest. These effects indicate that the app was more effective than the waitlist condition. Furthermore, wake after sleep onset, number of awakenings, PSQI, CES-D, and HADS improved and showed significant interaction effects (Table 4 and Table 5), but sleep onset latency, time in bed, terminal wakefulness, total sleep time, and DBAS-16 showed no significant effects at posttest. At follow-up, improvements on all outcome measures remained significant, except for number of awakenings.

**Table 3:** Demographic characteristics of participants in the Sleepcare mobile phone app and waitlist control conditions (n=151).

Characteristics		Group		Statistic	*P* value
		Waitlist (n=74)	App (n=79)		
**Age in years, mean (SD)**		41 (13.9)	39 (13.0)	*t*_149_=1.02	.31
**Sex, n (%)**				χ^2^_1_=0.13	.72
	Female	49 (64)	45 (61)		
	Male	28 (36)	29 (39)		
**Living together, n (%)**				χ^2^_1_=0.26	.61
	Yes	49 (64)	50 (68)		
	No	28 (36)	24 (32)		
**Employed, n (%)**				χ^2^_1_=0.65	.42
	Yes	56 (73)	58 (78)		
	No	21 (27)	16 (22)		
**Educational level, n (%)**				χ^2^_3_=1.10	.78
	Lower general secondary education	7 (9)	4 (5)		
	Higher general secondary education	10 (13)	9 (12)		
	Community college	11 (14)	9 (12)		
	University	49 (64)	52 (70)		
**Duration of insomnia in years, n (%)**				χ^2^_4_=5.40	.25
	<1	8 (10)	9 (12)		
	1-5	38 (49)	27 (36)		
	>5-10	10 (13)	13 (18)		
	>10	12 (16)	20 (27)		
	Unclear answer	9 (12)	5 (7)		
**Insomnia due to a physical condition, n (%)**				χ^2^_1_=0.20	.66
	Yes	9 (12)	7 (9)		
	No	68 (88)	67 (91)		
**Used sleep medication, n (%)**				χ^2^_1_=2.67	.10
	Yes	3 (4)	8 (11)		
	No	74 (96)	66 (89)		
**Prescribed sleep medication, n (%)**				χ^2^_1_=0.92	.35
	Yes	3 (100)	6 (75)		
	No	0 (0)	2 (25)		

**Table 4:** Multilevel analysis results of the diary and questionnaire variables for model 3: coefficients, standard errors, and *P* values^a^.

Variables			Intercept	Time	Condition	Interaction
**Diary variables**						
	**Sleep efficiency**					
		B	77.38	1.97	–0.24	5.34
		SE	0.78	0.99	1.11	1.55
		*P* value	<.001	.05	.83	.001
	**Sleep onset latency**					
		B	32.75	–3.59	0.12	–7.86
		SE	2.47	2.63	3.52	4.16
		*P* value	<.001	.18	.97	.06
	**Wake after sleep onset**					
		B	42.87	–3.74	3.17	–22.42
		SE	2.99	3.79	4.27	5.97
		*P* value	<.001	.33	.46	<.001
	**Number of awakenings**					
		B	2.20	–0.17	–0.11	–0.48
		SE	0.13	0.14	0.19	0.22
		*P* value	<.001	.21	.55	.03
	**Time in bed**					
		B	499.74	9.75	5.46	–16.07
		SE	5.02	5.22	7.16	8.28
		*P* value	<.001	.07	.45	.06
	**Terminal wakefulness**					
		B	36.93	–0.44	–1.50	–3.23
		SE	2.35	3.24	3.35	5.08
		*P* value	<.001	.89	.65	.53
	**Total sleep time**					
		B	387.6	18.6	2.76	14.9
		SE	5.53	6.18	7.89	9.77
		*P* value	<.001	.004	.73	.13
**Questionnaire variables**						
	**Insomnia Severity Index**					
		B	16.26	–3.04	0.20	–3.53
		SE	0.44	0.53	0.63	0.79
		*P* value	<.001	<.001	.75	<.001
	**Pittsburgh Sleep Quality Index**					
		B	10.50	–0.97	0.56	–2.52
		SE	0.33	0.36	0.47	0.54
		*P* value	<.001	.008	.23	<.001
	**Dysfunctional Beliefs and Attitudes** **about Sleep**					
		B	5.17	–0.34	0.18	–0.31
		SE	0.16	0.15	0.22	0.23
		*P* value	<.001	.03	.41	.18
	**Centre of Epidemiological Studies Depression scale**					
		B	14.95	0.66	1.54	–6.58
		SE	0.79	0.93	1.12	1.40
		*P* value	<.001	.48	.17	<.001
	**Hospital Anxiety and Depression Scale**					
		B	5.69	0.41	0.38	–2.36
		SE	0.35	0.43	0.50	0.65
		*P* value	<.001	.35	.45	<.001

^a^The covariates are not reported in this table.

**Table 5:** Multilevel analysis results of the diary and questionnaire variables: model comparisons^a^.

Variables			Models									
			0 vs 1		1 vs 2		2 vs 3		0 vs 3		4 vs 5	
**Diary variables**												
	**Sleep efficiency**											
		χ^2^_1_		23.23		1.53		11.54		36.30		18.66
		*R* ^2^		.07		.01		.03		.11		.04
		*P* value		<.001		.22		<.001		<.001		<.001
	**Sleep onset latency**											
		χ^2^_1_		9.83		0.39		3.57		13.79		12.21
		*R* ^2^		.02		.00		.01		.03		.01
		*P* value		.002		.53		.06		.003		<.001
	**Wake after sleep onset**											
		χ^2^_1_		14.82		0.62		13.29		28.73		11.99
		*R* ^2^		.05		.00		.03		.08		.02
		*P* value		<.001		.43		<.001		<.001		<.001
	**Number of awakenings**											
		χ^2^_1_		9.28		1.89		4.63		15.81		3.59
		*R* ^2^		.00		.01		.01		.02		.00
		*P* value		.002		.17		.03		.001		.06
	**Time in bed**											
		χ^2^_1_		0.69		0.03		3.82		4.54		5.38
		*R* ^2^		.00		.00		.01		.02		.01
		*P* value		.41		.85		.05		.21		.02
	**Terminal wakefulness**											
		χ^2^_1_		0.41		0.65		0.41		1.46		0.85
		*R* ^2^		.00		.01		.00		.01		.00
		*P* value		.52		.42		.52		.69		.36
	**Total sleep time**											
		χ^2^_1_		23.45		0.85		2.35		26.66		4.35
		*R* ^2^		.07		.00		.00		.08		.00
		*P* value		<.001		.36		.13		<.001		.04
**Questionnaire variables**												
	**Insomnia Severity Index**											
		χ^2^_1_		86.00		4.65		19.07		109.73		66.02
		*R* ^2^		.25		.02		.05		.31		.30
		*P* value		<.001		.03		<.001		<.001		<.001
	**Pittsburgh Sleep Quality Index**											
		χ^2^_1_		43.05		0.91		20.65		64.61		9.28
		*R* ^2^		.11		.01		.05		.15		.04
		*P* value		<.001		.34		<.001		<.001		.002
	**Dysfunctional Beliefs and Attitudes about Sleep**											
		χ^2^_1_		16.32		0.12		1.86		18.31		11.65
		*R* ^2^		.02		.00		.00		.02		.05
		*P* value		<.001		.07		.17		<.001		<.001
	**Centre of Epidemiological Studies Depression scale**											
		χ^2^_1_		8.34		0.96		20.67		29.97		18.66
		*R* ^2^		.02		.01		.05		.08		.08
		*P* value		.004		.33		<.001		<.001		<.001
	**Hospital Anxiety and Depression Scale**											
		χ^2^_1_		3.17		1.49		12.75		17.41		10.03
		*R* ^2^		.01		.01		.04		.06		.03
		*P* value		.07		.22		<.001		<.001		.002

^a^The covariates are not reported in this table.

Among the participants who completed the pre- and posttest, we found a clinically meaningful change on the ISI (∆ISI≥8) [40] between the waitlist and the app conditions. We also observed a significant clinically meaningful change 20 times in the app condition (20/45, 44%) and 7 times in the waitlist condition (7/62, 11%) at the posttest. In the app condition, significantly more people reached a meaningful clinical change (χ^2^_1_=15.19, *P*<.001). Before treatment, all participants had an ISI score >7 [26]. Of the participants who completed the posttest, 17 in the app condition (17/45, 38%) and 6 in the waitlist condition (6/62, 10%) had an ISI score ≤7. In the app condition, significantly more participants dropped below the insomnia threshold of ISI ≤7 than in the waitlist condition (χ^2^_1_=12.20, *P*<.001). At follow-up, 7 of the 29 participants had an ISI score ≤7 (7/29, 24%).

### Treatment Adherence

We divided treatment adherence into 4 components objectively measured by the app: (1) the number of sleep diaries filled out, (2) the number of relaxation exercises performed, (3) the number of conversations completed, and (4) the deviation between real time in bed and agreed-upon time in bed (Table 6). Only 2 of the 74 participants did not download the app. Furthermore, the adherence data showed different adherence patterns (see Multimedia Appendix 3). Most participants (n=35) filled out >35 diaries, 13 participants filled out <7 diaries, and the other 24 participants filled out between 7 and 35 diaries. This pattern follows a U-shaped curve. For the relaxation exercise, another pattern can be distinguished. More than half of the participants (n=41) performed a maximum of 7 relaxation exercises, of whom 11 participants did not do any relaxation exercises at all; 7 participants did >35 relaxation exercises. For the conversations the opposite was true: most participants (n=47) finished ≥90% of the conversations. Only a few participants (n=4) finished <10% of the conversations. A total of 38 participants started and committed to the sleep restriction exercise, meaning that they came to an agreement with the coach about their time in bed. Participants could stay in bed for longer or for less time than the agreement, and both situations occurred. Of these 38 participants, 32 stayed in bed for longer than agreed on for most of the nights involving sleep restriction. When participants stayed in bed too long it was by 67 minutes on average (SD 45); when they shortened their time in bed it was by 42 minutes on average (SD 29). Of the 38 participants, 26 (68%) were adherent, meaning that their time in bed deviated by an average of <60 minutes from the agreed-upon time in bed.

**Table 6:** Treatment adherence among 72 of 74 participants who downloaded the Sleepcare app.

Adherence component	Participants who performed the component, n	Proportion of the component completed, mean (SD)	Range	Participants with adequate dose, n (%)^a^
Number of diaries filled out	72	29.1 (16.4) times/49 (59%)	0-48 times	35/72 (49%)
Number of relaxation exercises performed	72	10.8 (12.0) times/49 (22%)	0-45 times	7/72 (10%)
Number of completed conversations in the training	72	83% (27%)	0%-100%	47/72 (65)
Deviation from sleep restriction in minutes	38	59.2 (46.4) minutes	9-285 minutes	26/38 (68)

^a^Adequate dose: diaries >35, relaxation exercises >35, deviation from sleep restriction <60 minutes, conversations >90%.

## Discussion

In this large-scale randomized controlled trial, we investigated the efficacy of CBT-I delivered via a mobile phone app. The results show that the app had moderate significant effects than in a waitlist on the primary measures of insomnia severity (*d*=–0.66, 95% CI –0.99 to –0.33) and sleep efficiency (*d*=0.71, 95% CI 0.37-1.04). The following secondary measures also improved compared with the waitlist: wake after sleep onset, number of awakenings, PSQI, depression, and anxiety. Other measures, such as time in bed, total sleep time, and dysfunctional beliefs about sleep did not show significant improvements, which could be explained by the exercises included in the treatment. The focus on modified sleep restriction in the app could explain the lack of improvement in time in bed and total sleep time, since sleep restriction aims at increasing sleep efficiency, starting by decreasing time in bed and thereby also influencing total sleep time. In addition, the app did not contain a cognitive exercise, which could explain why we found no difference in the dysfunctional beliefs about sleep. Nevertheless, at posttest, 44% of the participants in the app condition had achieved a clinically meaningful change compared with 11% in the waitlist condition. The improvements were largely sustained at the 3-month follow-up. The observed effects on the primary measures are similar to those reported in a recent meta-analysis on sleep efficiency (Hedge *g*=0.58) and somewhat lower (but in the same range) than those reported for insomnia severity (Hedge *g*=1.09) [16]. Note that the meta-analysis was based on studies with various levels of human involvement, ranging from no human support to personal contact as part of the intervention. Earlier research indicated that human support increases efficacy [55]. However, the effect sizes in our study were achieved without any form of human support.

Regarding automated support, this study most closely resembles the trials by Espie and colleagues [48] and Ritterband and colleagues [56], which both offered automated Web-based CBT-I. These Web-based treatments packaged the full scope of CBT-I and demonstrated large effects. Espie and colleagues found a Cohen *d* of 0.95 for sleep efficiency. Ritterband and colleagues found a Cohen *d* of 1.26 for insomnia severity and 0.68 for sleep efficiency. Again, the observed effect sizes in our study were more or less in the same range as these published results, and our effects were achieved without including the full CBT-I package (eg, we did not include cognitive therapy and stimulus control). The app concentrated on sleep restriction, and as a result the effects for sleep efficiency are more pronounced than those for insomnia severity. The focus on sleep restriction may also explain the absence of an effect on total sleep time.

Zachariae and colleagues [16] found in their meta-analysis that 58.7% to 100% of the participants in the CCBT-I conditions completed postintervention assessments, with an average of 75.3%. In our study, 61% of the participants in the app condition completed postintervention assessment questionnaires, while 81% of the participants in the waitlist condition did so. This difference can probably be explained by the fact that the participants in the waitlist received the app only after they had filled out the postintervention assessment. However, the number of participants filling out assessments may not necessarily correspond to the number of participants who complete interventions. Therefore, we also report treatment adherence numbers and adequate doses. Previously, Espie and colleagues [48] found that 88% of their participants received an adequate dose (≥4 sessions). Lancee and colleagues [55] found that 83% received an adequate dose of the modules in the support condition, and 60% in the no-support condition. In our trial, adherence was measured for the different components, with adherence rates ranging from 10% to 68%. Apart from the relaxation exercise adherence (where only 10% of the participants received an adequate dose), the other adherence rates are comparable with the 60% found by Lancee and colleagues [55] in their no-support condition.

In general, there exists a positive relationship between treatment adherence and treatment outcome [50]. This relationship suggests that this trial could have been more successful if relaxation adherence rates would have been higher, for example. Relaxation exercises have been noted as an effective treatment as part of multicomponent treatment [36]. Therefore, it is likely that higher adherence rates to the relaxation exercise would have improved the overall efficacy of the app. However, relaxation exercises as stand-alone treatment were inferior to other CBT-I exercises, such as stimulus control and sleep restriction [12]. So, as in all multicomponent treatments, it remains unclear how adherence to specific components affected the overall efficacy of the app. In this trial, overall adherence rates were adequate, but there was also a considerable number of people who did not start the modified sleep restriction exercise at all. Beforehand, we decided that it was better to keep people in no or a suboptimal sleep restricting schedule rather than letting them drop out of the treatment altogether. A possible risk of suboptimal treatments could be that people may not seek further help after a less-successful treatment. However, this is a general health care risk, which is not typical for eHealth or self-help interventions. Nevertheless, the optimal tradeoff between individual autonomy and strictness in mobile phone app regimens has yet to be determined in future studies.

### Limitations and Future Work

This study has a number of limitations that should be considered in relation to the findings. Since the goal of the study was to demonstrate the efficacy of the app first in a group with insomnia disorder but without too much sleep impairment, we used an ISI score of >7, meaning that we excluded people who slept <5 hours as measured by a sleep diary. This exclusion criterion may have led to a floor effect and the inclusion of participants with relatively little room for improvement. Although it is hard to compare studies because of different inclusion criteria, it seems that Espie and colleagues [48] only included participants with more severe insomnia (baseline sleep efficiency of 55%–65%). It may be possible to achieve larger effects in samples with higher levels of symptoms. However, it remains the case that the efficacy of our mobile phone app has not yet been demonstrated in a sample with severe insomnia. Because this was one of the first times a stand-alone app has been used to deliver CBT-I, we also excluded participants with comorbidities such as depression. This and the issues mentioned above limit the generalizability of our results, especially given the high comorbidity of depression and insomnia. Now that the app has proven its efficacy in a relatively mildly affected sample, future research could expand the inclusion criteria (eg, severe insomnia, depression) to study the efficacy of a CBT-I app in a more severely affected population.

A methodological limitation was that no other Web-based or face-to-face treatment group was included. Several other studies have already demonstrated the efficacy of CCBT-I and CBT-I programs. However, a similar Web-based condition could provide insight into the added value of a mobile app. Another related limitation is that there was no placebo control group. It may very well be that nonspecific factors played a role in the treatment effects of the app. Other methodological limitations were that this study used self-report measures, and polysomnography would be needed to confirm the objective changes in sleep. Furthermore, the participants in this study were a self-selected sample and may have been an unusual group of people who were interested in solving their sleep difficulties with self-help. A high percentage of the sample consisted of university-educated participants, who represent only a part of society.

Another limitation was that the app focused on sleep restriction and relaxation. Future work should include more of the other CBT-I components, such as cognitive exercises, and evaluate those. Mobile phone apps provide us with the unique opportunity to study the separate components of CBT-I in a controlled way. Future research could focus on studying the separate components, so that more insight can be gained into the individual efficacy of these CBT-I components. In addition, future research should compare the efficacy of the classical sleep restriction versus the modified sleep restriction exercise.

Lastly, there were some technical issues during the randomized controlled trial that made it impossible for some participants to continue to the next conversation. The occurrence of this problem was monitored and solved when needed. In these cases, a new conversation was manually planned in the database for a specific participant, and an email with instructions to update the app was sent to that participant.

### Conclusion

We are confident that this study has produced insights into the domain of automated e-coaching apps for insomnia. These apps provide an opportunity to investigate separate treatment components while minimizing the influence of nonspecific therapist factors such as therapeutic alliance. Keeping the limitations in mind, this study demonstrated the efficacy of a mobile phone app in the treatment of insomnia. These effects were clinically meaningful and in the range of what is found for Web-based treatment in general. This supports the applicability of these kinds of technical tools in the treatment of insomnia. Through these apps, many more people can be offered effective insomnia treatment with probable reduced costs. We are confident that mobile phone apps will prove to be useful in the realm of prevention treatments; it remains to be determined how they should best be offered: in a stand-alone format for (prevention) treatment, or within a blended care framework where the sleep specialist uses an app to improve and accelerate insomnia treatment.

## Appendix

Multimedia Appendix 1 CONSORT-EHEALTH checklist V1.6.1 [25].

Multimedia Appendix 2 Pretest questionnaire.

Multimedia Appendix 3 Adherence patterns.

## Abbreviations

CBT-I: cognitive behavioral therapy for insomnia
CCBT-I: computerized cognitive behavioral therapy for insomnia
CES-D: Centre for Epidemiological Studies Depression
DBAS-16: brief Dysfunctional Beliefs and Attitudes about Sleep
DSM-5: Diagnostic and Statistical Manual of Mental Disorders
HADS: Hospital Anxiety and Depression Scale
ISI: Insomnia Severity Index
PSQI: Pittsburgh Sleep Quality Index
