# Supplementary material for: Mobile Phone-Delivered Cognitive Behavioral Therapy for Insomnia: A Randomized Waitlist Controlled Trial
Source: J Med Internet Res. 2017 Apr 11;19(4):e70. doi: 10.2196/jmir.6524 (PMC5405291; doi:10.2196/jmir.6524)
Supplement: Multimedia Appendix 2 [file jmir_v19i4e70_app2.pdf]

Questions that were included in the online pre-test questionnaire to verify the inclusion and exclusion criteria:

- Are you older than 18 years?
- What is your date of birth?
- Do you have an Android phone? (e.g. Samsung, HTC, LG)
    - What brand of phone do you have?
    - Do you have Android version 4.1 or higher (eg. 4.4.2)? You can find this in your settings on your phone.
- What is your gender?
- What is your highest level of completed education?
- Do you currently have a paid job?
- Do you work in shifts?
- Do you live together with another person?
- Are you currently pregnant or breast-feeding?
- How long have you been bothered by your chronic insomnia? (years/months)
- Does your chronic insomnia negatively affect your daytime functioning (fatigue, ...
- On average, how many nights a week do you suffer from your insomnia?
- How many days per month do you drink three or more glasses of alcohol per day?
- How often do you smoke marijuana / hashish / marijuana?
- Do you currently suffer from schizophrenia and/or psychosis?
- Are you currently being treated by a psychologist / psychiatrist?
    - When did you start with this treatment?
- Did you seek help with your sleep problems before? If yes, what have you tried? You can select multiple options
    - No previous help
    - Sleep medications
        - How satisfied were you with the sleep medication?
        - Could you explain this (optional)?
    - Treatment for my sleep problem through (online) self-help therapy
        - How satisfied were you with self-help through an online program?
        - Could you explain this (optional)?
    - Treatment for my sleep problem by a psychologist
        - How satisfied were you with the guidance of a psychologist?
        - Could you explain this (optional)?
    - Otherwise, namely:
- Does your insomnia have a clearly identifiable physical cause, such as pain?
    - If yes, you can then (briefly) describe the physical cause?
- Do you use drugs? Either for your sleep problems or other symptoms.
    - I use medication to sleep
    - I use medication for psychological complaints other than my insomnia, eg anxiety and depression
    - I use other medications
- Do you take prescription medication (prescribed by a doctor) or freely available medication?
- Did the medication, dose and/or frequency changed in the last six weeks?
    - If yes, write down the name, dose and timing of taking your (sleep) medication.

These questions were complemented by the Insomnia Severity Index [25], a subscale of the SLEEP-50 [26] to determine possible sleep apnoea, and a subscale of the CES-D [27, 28] to determine symptoms of depression. Furthermore, the following questionnaires were also part of the pre-test as secondary outcome measures: PSQI [41], DBAS [42], and HADS [43, 44].
