# Supplementary figures and images for: Mobile Phone-Delivered Cognitive Behavioral Therapy for Insomnia: A Randomized Waitlist Controlled Trial
Source: J Med Internet Res. 2017 Apr 11;19(4):e70. doi: 10.2196/jmir.6524 (PMC5405291; doi:10.2196/jmir.6524)

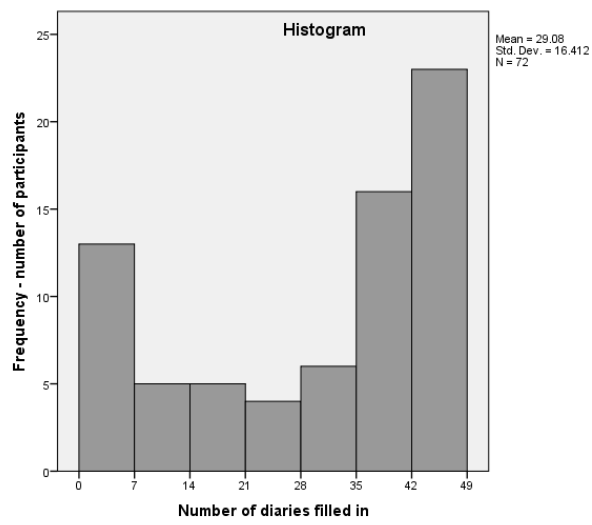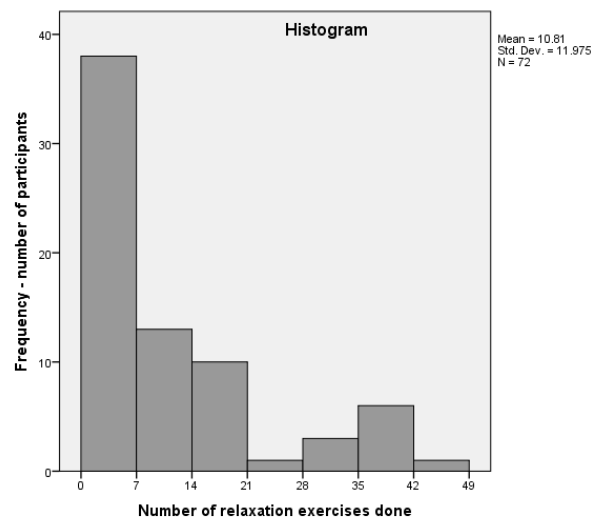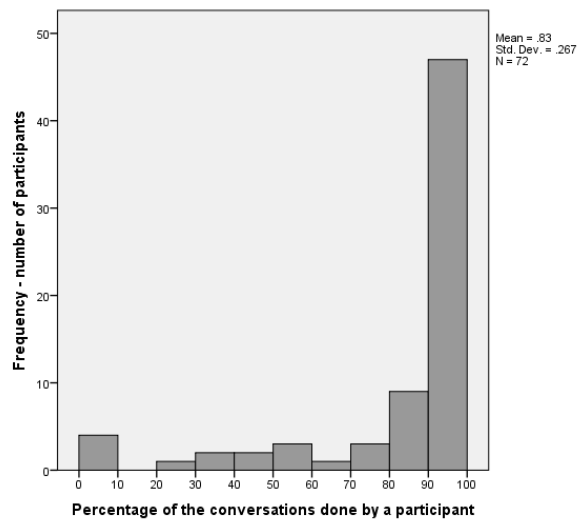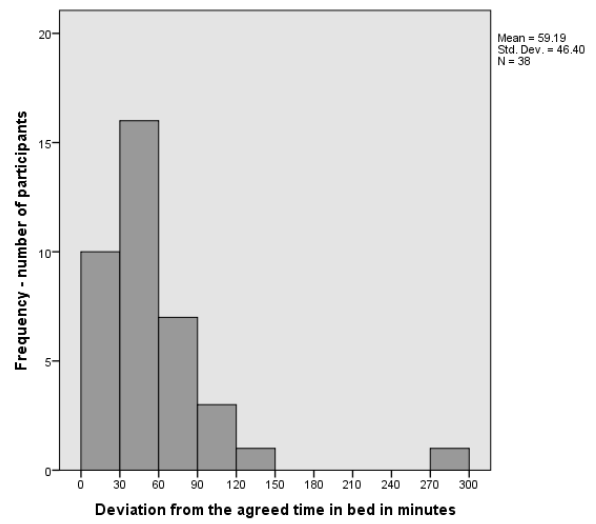

Supplement: Multimedia Appendix 3 [file jmir_v19i4e70_app3.pdf]
